# Parliament and the Professions

**Published:** 1920-04-03

**Authors:** 


### Parliament and the Professions.

### Miners' Nystagmus.

Replying for the Home Secretary, Major Baird informed Mr. Swann that in 1914 (the last year for which figures are available) there were 5,992 cases of miners' nystagmus in which compensation was paid to miners on account of this disease; 3,218 of these cases were continued from previous years. Working in the insufficient light given by miners' lamps is recognised as the main cause, and the question of improving the illumination is being investigated by a Departmental Committee. A series of practical trials with lamps giving a better light is about to be carried out.

### Council under the Nurse Registration Act.

Mr. Grundy asked the Minister of Health whether he can give an assurance that in the appointment of the first Council under the Nurse Registration Act there will be included representatives directly nominated by *bona fide* nurses' trade unions, as distinct from associations presumed to cater for nurses, but directed and controlled by persons other than nurses.

Dr. Addison replied that under the schedule to the Act he is bound to consult, and he has consulted, three organisations specifically named, and such other associations or organised bodies of nurses or matrons as ask to be consulted. No organisation is given the right of direct nomination to the General Nursing Council.

### Uncertifiable Cases amongst Discharged Men.

Sir A. Shirley Benn asked the Pensions Minister for information as to sanatoria for the benefit of uncertifiable border-line cases of loss of mental balance occurring among ex-soldiers as distinguished from neurasthenics of a pseudo-paralytic type.

Sir James Craig replied that there are eighteen institutions, providing 2,046 beds, for these cases, and further institutions are in course of preparation.

